# Microfibril orientation and compositional heterogeneity in fiber and vessel cell walls of poplar xylem studied by AFM-IR and SFG spectroscopy

**DOI:** 10.1007/s00425-026-04947-7

**Published:** 2026-02-19

**Authors:** Jongcheol Lee, Juseok Choi, Yen-Ting Lin, Fangxin Qian, Botong Tong, Quanzi Li, Seong H. Kim

**Affiliations:** 1 Department of Chemical Engineering and Materials Research Institute, Pennsylvania State University, University Park, PA 16802 USA; 2 Institute of Industrial Crops, Heilongjiang Academy of Agricultural Sciences, Harbin, 150086 China; 3 National Key Laboratory for Development and Utilization of Forest Food Resources; Key Laboratory of Forest Genetics and Breeding, International Research Center for Plant Cell Wall, College of Forestry and Biotechnology, Zhejiang A&F University, Hangzhou, 311300 China

**Keywords:** Microfibril angle (MFA), Photothermal AFM-IR, Plant cell wall structure, Sum frequency generation (SFG) microscopy, Secondary cell wall, Xylem

## Abstract

**Main conclusion:**

This study demonstrates the use of photothermal AFM-IR and vibrational SFG microscopy to investigate nanoscale chemical heterogeneity and mesoscale cellulose microfibril orientation in hybrid poplar xylem, revealing differences in cellulose microfibril (CMF) orientation between fiber and vessel cell walls that are consistent with their mechanical support and hydraulic functions.

**Abstract:**

Understanding the structural organization of cellulose microfibrils (CMFs) within individual plant cell walls is essential for connecting cell wall architecture to its mechanical and physiological functions. However, due to the complex hierarchical structure and nanoscale heterogeneity of cell walls, it remains technically challenging to resolve detailed compositional and orientational information at subcellular levels of individual cell walls. This study investigates the internal 3D structure, chemical composition, and sublayer organization of fiber and vessel cell walls in the xylem tissue of a two-year-old field-grown hybrid poplar tree (*Populus alba* × *P. glandulosa*) using photothermal atomic force microscopy coupled with infrared spectroscopy (AFM-IR) and sum frequency generation (SFG) hyperspectral microscopy. AFM-IR provided nanoscale chemical imaging, revealing localized compositional heterogeneity, including variations between adjacent cell walls and transitional layers beyond the traditional S1, S2, and S3 sublayers. SFG microscopy revealed that CMFs in fiber walls are highly aligned along the stem axis, consistent with their role in mechanical support, while vessel cell walls exhibited slightly tilted CMFs, reflecting their function in hydraulic transport. Together, these results offer new insights into cell-type-specific CMF organization and compositional gradients in hybrid poplar xylem. These findings highlight the structural and chemical complexity of secondary cell walls in woody plants and demonstrate the value of AFM-IR and SFG spectroscopy in elucidating plant cell wall architecture.

**Graphical abstract:**

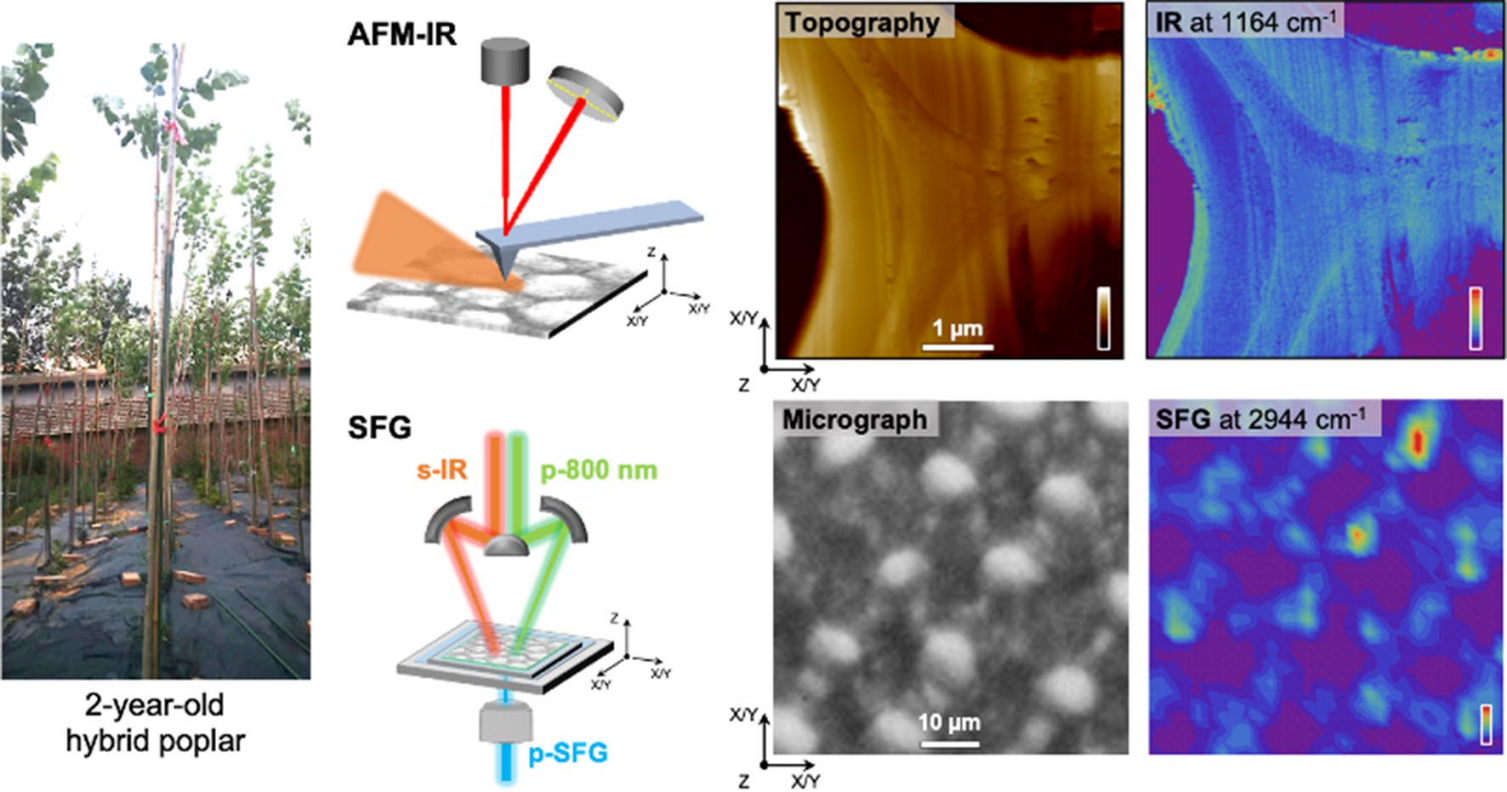
.

**Supplementary Information:**

The online version contains supplementary material available at 10.1007/s00425-026-04947-7.

## Introduction

**Fig. 1:**
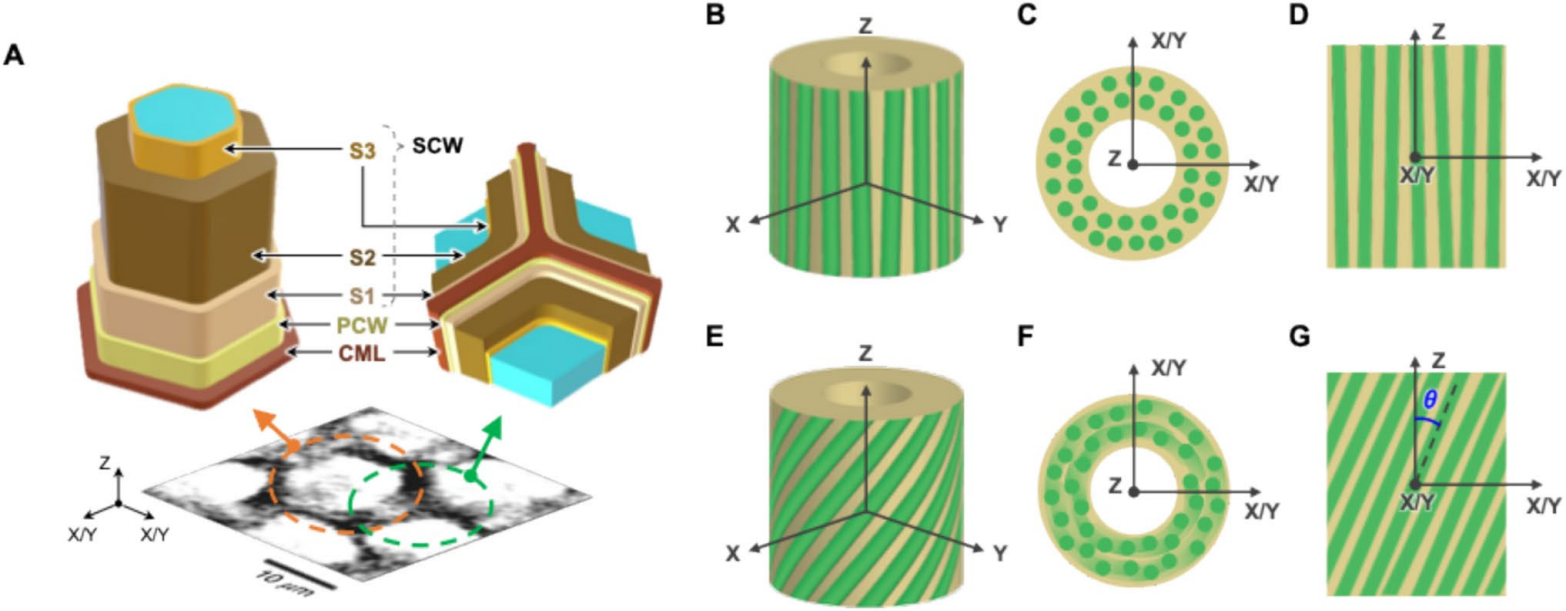
**A-G** Schematic illustration of the layered structure of a plant cell wall (**A**) and schematic representation of cellulose microfibril orientations in different views (**B-G**). CML, PCW, and SCW represent the compound middle lamella, primary cell wall, and secondary cell wall, respectively. **B-G** The stem or cell long axis was aligned along the Z-axis. The tilt angle (θ) refers to the deviation of microfibrils from this Z-axis, and the azimuth angle (ϕ) refers to the angles from the X or Y-axis within the XY plane (not shown here). **B-D** Microfibrils aligned along the Z-axis, where the tilt angle is close to zero. **E–G** Microfibrils tilted from the Z-axis, with a distinct tilt angle (θ). The transverse cross-sections (**C**, **F**) are cuts along the XY plane. The color fading in **F** represents microfibrils that are gradually buried beneath the cross-sectional surface due to the tilt angle. The longitudinal cross-sections (**D**,** G**) are cuts along the ZX or ZY planes. The polarity (directionality) of individual microfibrils is not represented in these diagrams

Cellulose is a linear homopolymer composed of β-D-glucose units connected through β(1,4)-glycosidic bonds (French 2017). Cellulose microfibrils (CMFs) are synthesized by cellulose synthase complexes (CSCs) in the plasma membrane (Hill et al. 2014; Nixon et al. 2016; Purushotham et al. 2020). These complexes, composed of multiple cellulose synthase (CESA) proteins, extrude cellulose chains into the cell wall where they aggregate to form microfibrils (Taylor et al. 2003; Li et al. 2016; Nixon et al. 2016). These microfibrils can further bundle into larger crystalline domains. Primary cell walls (PCWs), which are formed during cell expansion, are typically thin and flexible layers, and contain loosely bundled, relatively disordered CMFs (Zhang et al. 2016). In contrast, secondary cell walls (SCWs) are deposited after cell growth ceases and contain aligned and bundled CMFs (Crowe et al. 2021). SCWs are thicker, mechanically reinforced, and known to be composed of three distinct layers—S1, S2, and S3—each with different microfibril orientations (Fig. 1A) (Zhong and Ye 2014; Reza et al. 2019). The S2 layer, in particular, is the thickest and plays a dominant role in determining mechanical properties such as stiffness and tensile strength (Donaldson and Xu 2005).

Cellulose plays a crucial role in plant structure and growth (Cosgrove 2005; Somerville 2006). In woody plants, the arrangement of CMFs in secondary cell walls significantly influences wood properties and functions (Lachenbruch and McCulloh 2014). Figure 1B–D illustrates vertically aligned CMFs with zero tilt angle, whereas Fig. 1E–G shows CMFs with a tilt angle. The microfibril angle (MFA), which is the tilt angle between the cellulose microfibrils and the longitudinal axis of the cell (θ in Fig. 1G), is a key structural parameter that affects wood strength, stiffness, and other mechanical properties (Barnett and Bonham 2004; Lachenbruch and McCulloh 2014). Accurate characterization of CMF structures, particularly MFA, is essential not only for fundamental plant science but also for a wide range of applications, including the design of engineered wood products (Gherardi Hein and Tarcísio Lima 2012; Wang et al. 2024). Compositional and structural features, such as MFA and microfibril organization, impact the mechanical integrity, processability, and recalcitrance of lignocellulosic biomass, making them critical parameters in industrial biotechnology (Abramson et al. 2010; Spence et al. 2010; de Souza et al. 2013; DeMartini et al. 2013; Silveira et al. 2013; Wu et al. 2014; Zhang et al. 2021).

Recent studies have characterized cellulose microfibril structures in poplar xylem using a combination of genetic, spectroscopic, and imaging techniques. Poplar (*Populus* spp.) is widely used as a model woody species due to its fast growth, sequenced genome, and well-developed genetic tools (Tuskan et al. 2006). It also exhibits well-defined secondary growth, allowing detailed studies of xylem structure and wood formation (Tuskan et al. 2006; He et al. 2022). Research has shown that specific cellulose synthase genes such as *CesA4*, *CesA7/17*, and *CesA8/18* are essential for secondary wall formation in poplar, with knockdown resulting in reduced cellulose content, altered xylem structure, and compromised mechanical properties (Abbas et al. 2019; Xu et al. 2021). Recent work has demonstrated that overexpression of a gibberellin 20-oxidase gene (*AtGA20ox1*) in xylem tissue alters cellulose properties, increasing crystal size and enhancing the bundling of cellulose microfibrils (Peng et al. 2023). When these cellulose crystals were incorporated into paper materials, improved mechanical properties were obtained, suggesting that gibberellin-mediated regulation can be leveraged to engineer nanocellulose for value-added applications (Peng et al. 2023). These findings highlight the intricate regulation of CMF formation and organization, with consequences for cell wall strength, vascular function, and biomass quality.

Various characterization techniques have been employed to study cellulose microfibril (CMF) structure in poplar xylem cell walls. X-ray diffraction (XRD) has been used to determine cellulose crystallinity and crystal size, providing bulk structural information about cellulose organization (Peng et al. 2023). Scanning electron microscopy (SEM) enables morphological visualization of fiber and vessel wall architecture, including measurements of wall thickness and fiber structure (Abbas et al. 2019; Hao et al. 2024). Atomic force microscopy (AFM) provides high-resolution surface topography and has been used to observe the size of bundled cellulose crystals (Peng et al. 2023). However, these traditional techniques often require dry or pre-treated samples and may lack the ability to spatially resolve chemical and structural features of cell walls in intact, hydrated tissues.

Polarized light-based techniques have also been used to probe CMF orientation in plant cell walls. Retardation imaging using polarized optical microscopy (POM) enables semi-quantitative MFA values across different cell types, while the lack of precise information about cross-sectional thickness and variation in cellulose content across wall sublayers limits its quantitative accuracy (Kita et al. 2022, 2023). Polarized infrared (IR) microscopy has been used to probe the molecular orientation of cell wall polymers. Studies by Salmén and colleagues demonstrated anisotropic alignment of cellulose and lignin from individual cell walls, offering complementary insights into cellulose organization and microfibril orientation (Simonović et al. 2011; Peng et al. 2019). However, IR is limited by a coarse effective spatial resolution—nominally ~ 25 µm in point mode but larger in practice due to diffraction-induced Gaussian tails—and by the intrinsic “magic-angle” condition that can make dichroism indistinguishable from random orientation (Kafle et al. 2017).

Vibrational and nonlinear optical techniques offer chemically specific, high-resolution mapping capabilities. Polarized Raman imaging enables chemically specific, high-resolution mapping of MFA at the subcellular level, allowing layer-specific analysis within secondary cell walls (Gierlinger et al. 2009; Sun et al. 2016). However, its application is limited by the need for well-prepared flat sections and reduced signal quality in highly lignified layers due to fluorescence background. Second-harmonic generation (SHG) microscopy has also been applied for MFA study in secondary cell walls with submicron resolution (Brown et al. 2003; Richely et al. 2023). However, these studies have provided only qualitative information on microfibril orientation projected in the imaging plane.

Several studies have employed photothermal atomic force microscopy coupled with infrared spectroscopy (AFM-IR) to investigate the nanoscale chemical composition and structural heterogeneity of plant cell walls. Bilkey et al. (2022) mapped mechanochemical properties of primary walls in *Arabidopsis* epidermal cells, revealing that local carbohydrate composition at cell–cell junctions correlates with increased stiffness. Pereira et al. (2018) analyzed pit membranes in poplar xylem and identified chemically distinct zones enriched in proteins, lignin, and polysaccharides, highlighting spatial heterogeneity. Coste et al. (2021) examined the effects of resin embedding on poplar (*P. tremula* × *P. alba*) stem cross-sections and found that resin infiltration can alter both IR spectral features and mechanical stiffness across wall sublayers. Kesari et al. (2021) applied photo-induced force microscopy (PiFM) to spruce wood and discovered nanometer-scale, lignin-depleted domains within the secondary wall as well as orientation-dependent IR responses.

In this study, we explored the use of AFM-IR and vibrational SFG hyperspectral microscopy to investigate the composition and MFA in the cell walls of xylem tissue from hybrid poplar (*P. alba* × *P. glandulosa*), which has been used as a model woody species in genetic engineering and plant biology research (Abbas et al. 2019; Peng et al. 2023; Hao et al. 2024). Our study provides nanoscale chemical imaging maps at multiple IR wavenumbers across fiber and vessel cell walls of field-grown hybrid poplar, prepared without the use of fixatives or resin. AFM-IR intensity ratio maps and principal component analysis (PCA) were used to more clearly identify wall sublayers and classify spectra, supporting the presence of compositional heterogeneity across the wall. In parallel, hyperspectral SFG analysis provided the CMF orientation in the secondary cell walls averaging over the sublayers. Together, these techniques enabled a multiscale investigation of the structural and chemical organization of the secondary cell walls in hybrid poplar, facilitating future nanoscale studies of gene-induced changes in specific wall sublayers.

## Materials and methods

### Sample preparation

**Fig. 2:**
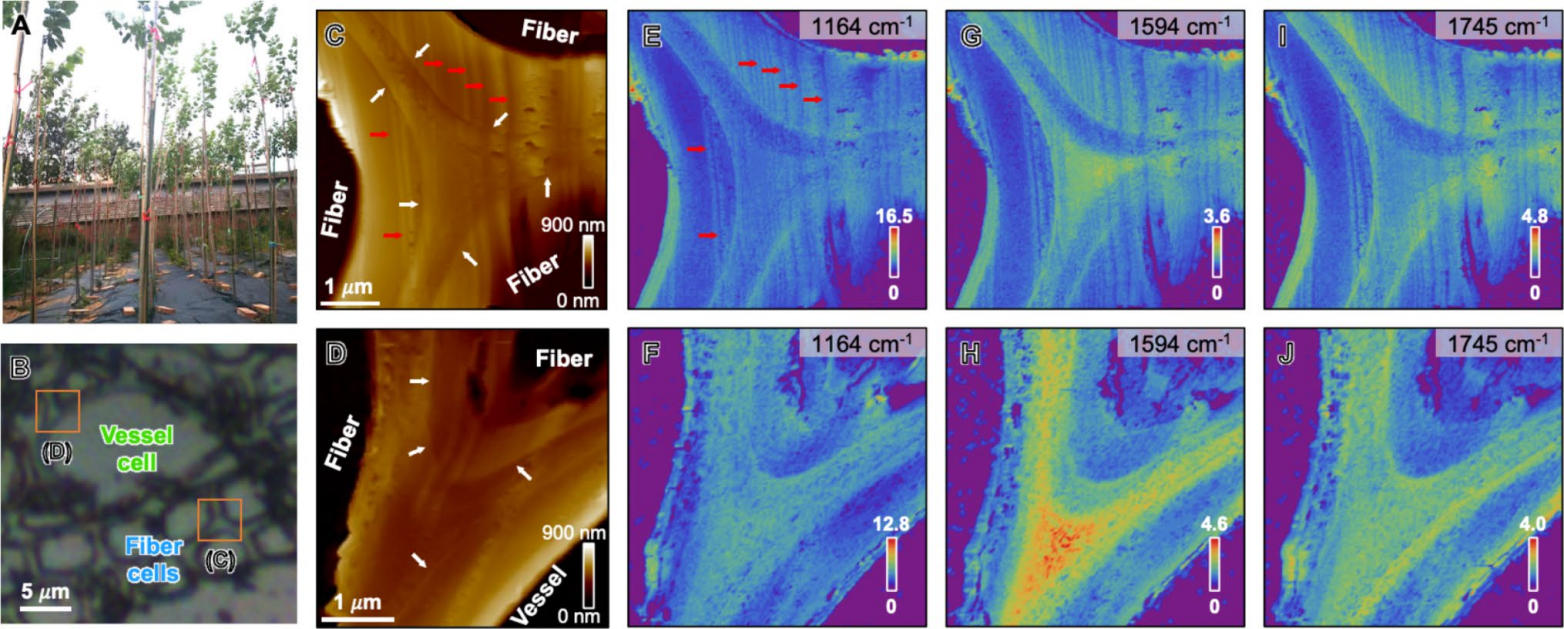
**A-J** AFM-IR study of cross-sectioned poplar xylem cell walls. **A** Two-year-old, field-grown hybrid poplar trees (*P. alba* × *P. glandulosa*). **B** Micrograph of a transversely cross-sectioned poplar xylem tissue. Boxed areas indicate the regions selected for AFM-IR analysis. **C–D** AFM-IR topography images of cell walls surrounded by fiber cells (**C**) and vessel and fiber cells (**D**). The areas labeled ‘Fiber’ and ‘Vessel’ in the topography maps corresponds to the lumens of fiber and vessel cells. **E–J** AFM-IR amplitude maps at 1164 cm⁻1 (**E**, **F**), 1594 cm⁻1 (**G**, **H**), and 1745 cm⁻1 (**I**, **J**)

A two-year-old, field-grown wild-type hybrid poplar (*P. alba* × *P. glandulosa*) tree grown at the Chinese Academy of Forestry (Beijing, China) was used for this study (Fig. 2A). The tree was harvested in July, and the diameter of the basal stem was ~ 20 mm. Bottom stem sections of the poplar tree (~ 20 mm in diameter) were stored in 70% isopropyl alcohol/water after removing bark. The stem section was further cut into small pieces (~ 5 mm), washed in distilled water, and then underwent additional sectioning for analysis. Regions of developed earlywood cell walls within the second annual ring were analyzed. For SFG analysis of transversely sectioned samples, the small pieces were sectioned to 20 μm thickness using a Leica CM1950 cryostat with Shandon™ Cryomatrix™ (Thermo Fisher Scientific). The sectioned samples were carefully washed with water, which was then fully replaced with D_2_O to prevent IR attenuation in the OH stretch region by H_2_O. These samples were then sandwiched between a cover glass and a slide glass with D_2_O in between. Multiple cell walls from three transverse sections, derived from the same tree, were examined for SFG analysis.

Additional longitudinal sections (about 100 μm thick) were prepared using the same method, except that the small stem pieces were boiled for 30 min to facilitate sectioning (Tardif and Conciatori 2015). The softening step has been reported to cause minimal compositional changes under short exposure (Cohen and Harris 1937). These longitudinal sections were then air-dried on glass slides and used for SFG analysis and wide-angle X-ray scattering (WAXS). WAXS data were collected using a XENOCS Xeuss 2.0 system equipped with a Cu x-ray source (λ = 1.5418 Å). Two longitudinal sections were examined for SFG analysis, and one sample was used for WAXS.

Additional ultrathin samples were transversely sectioned using a Leica EM UC6 ultramicrotome for photothermal AFM-IR analysis. The stem pieces were fixed in 2.3 M sucrose, ultra-microtomed into ~ 1 μm-thick sections using a glass knife, and mounted on a gold-coated glass slide prepared by gold sputtering. The sucrose matrix was then washed off with DI water, and the samples were air-dried. Two tricellular junction walls from the earlywood region of the second annual ring**—**one surrounded by three fiber cells and the other surrounded by a vessel cell and two fiber cells**—**were analyzed (Fig. 2C,D).

### Photothermal AFM-IR

The photothermal AFM-IR analysis was conducted using a Bruker IconIR system in contact mode, covering the spectral range of 800–1800 cm^−1^. A gold-coated AFM probe (PR-U-CnIR; spring constant k = 0.2 N m^−1^; resonant frequency = 13 kHz) maintained contact with the sample surface during scanning while the sample was illuminated by IR pulses from a MIRcat™ source. The IR pulse frequency was modulated to match the contact resonance frequency of 730 kHz, and the IR-induced oscillation signal was recorded at the same frequency, followed by normalization using the IR background spectrum. The four tilt mirrors, which guide the IR beams from four QCL chips (covering 780–992 cm^−1^, 992–1212 cm^−1^, 1212–1433 cm^−1^, and 1433–1810 cm^−1^), were initially calibrated using a PMMA reference sample at 987, 1145, 1257, and 1730 cm^−1^. Subsequent fine alignment was conducted on cell wall regions at 897, 1164, 1260, and 1745 cm^−1^, which are common IR peaks of cellulosic materials. This resulted in higher signals than the calibration with PMMA, likely due to the mechanical stiffness difference between dried cell walls and PMMA. The phase-locked loop (PLL) tracking was enabled with a ± 40 kHz frequency-shift window to accommodate contact resonance shifts caused by local nanomechanical variation. AFM-IR amplitude ratio maps were processed and visualized using a custom script written in *Mathematica*. The AFM-IR spectra were pretreated to correct discontinuity–abrupt intensity changes near 992, 1212 and 1433 cm^−1^ due to QCL chip switching–using a custom Python script. The spectral regions within ± 5 cm^−1^ of these switching points were excluded from principal component analysis (PCA).

### SFG microscopy system

SFG data were collected using a custom-designed SFG microscopy system. In the system, 800 nm pulses (85 fs pulse duration) were generated with a Ti–Sapphire amplifier (Libra; Coherent, Santa Clara, CA, USA) at a 2 kHz repetition rate. Tunable broadband IR pulses (100–150 cm^−1^ full width at half maximum) were generated with an optical parametric generator/optical parametric amplifier (OPG/OPA) system (OPerA Solo, Coherent). These two beams were spatially and temporally overlapped through a microscope, resulting in a few micrometers spatial resolution: 2.4 μm × 4.1 μm with a 36 × reflective objective and 5.4 μm × 7.9 μm with a 15 × reflective objective (Huang et al. 2018). A *pps* polarization combination was used; the letters represent the polarization (*p* or *s*) of the SFG beam emitted, incident 800 nm pulse, and incident IR pulse, respectively, with respect to the incidence plane. The transversely sectioned samples were analyzed in transmission mode, while the longitudinally sectioned samples were analyzed in reflection mode. The SFG data for hyperspectral imaging were collected by step-scanning the sample with a 2 μm step size using a 36 × objective, using two broadband IR beams centered at 2950 cm^−1^ for the CH stretch region and 3300 cm^−1^ for the OH stretch region.

## Results

### Photothermal AFM-IR analysis of hybrid poplar xylem

#### Unprocessed AFM-IR amplitude maps

Photothermal AFM-IR was employed to investigate the chemical composition and sublayer organization in the poplar xylem cell walls (Fig. 2A,B). The topography images (Fig. 2C,D) showed sublayer-like contrasts in cell walls (marked with white arrows). The AFM-IR amplitude maps were recorded at 1164 cm^−1^, 1594 cm^−1^, and 1745 cm^−1^ (Fig. 2E-J). The 1164 cm^−1^ peak is commonly attributed to C–O–C stretching in glycosidic linkages of cellulose, and 1745 cm^−1^ is attributed to C = O stretching in esterified pectin and hemicellulose (Wilson et al. 2000; Manrique and Lajolo 2002; Lee et al. 2015; Kafle et al. 2017; Coste et al. 2021). The 1594 cm^−1^ peak is putatively assigned to lignin (Agarwal and Ralph 1997; Coste et al. 2021). While these assignments have been used frequently in the literature, it is noted that they are not strictly unique and may be influenced by vibrational coupling, spectral overlap, and background contributions (Makarem et al. 2019). The raw intensity maps of AFM-IR provide additional sublayered features that are not obvious in the topography images, reflecting spatial distribution of chemical components (Fig. 2E-J). For example, a strong 1594 cm^−1^ intensity at the center of the three-cell junctions, and localized regions of reduced 1164 cm^−1^ intensity were also present within individual cell wall layers.

#### AFM-IR amplitude ratio maps with internal reference

**Fig. 3:**
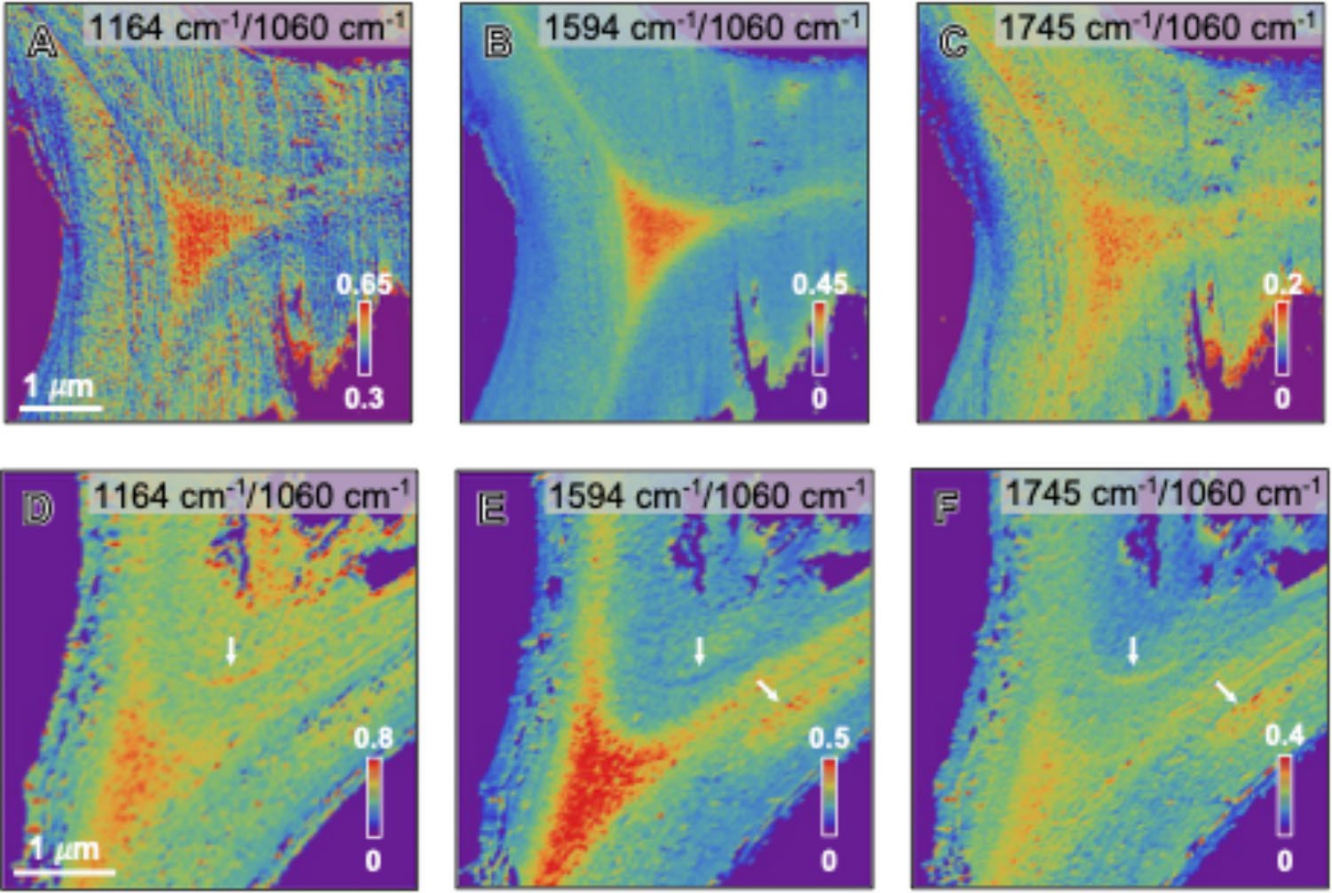
**A-F** AFM-IR amplitude ratio maps of 1164 cm⁻1/1060 cm⁻1 (**A**, **D**) and 1594 cm⁻1/1060 cm⁻1 (**B**, **E**), 1745 cm⁻1/1060 cm⁻1 (**C**, **F**) across cell walls of hybrid poplar. **A-C** Cell wall surrounded by fiber cells. **D-F** Cell walls surrounded by vessel and fiber cells. Scale bars in **A** and **D** apply to **B** and **C,** and **E** and **F**, respectively

**Fig. 4:**
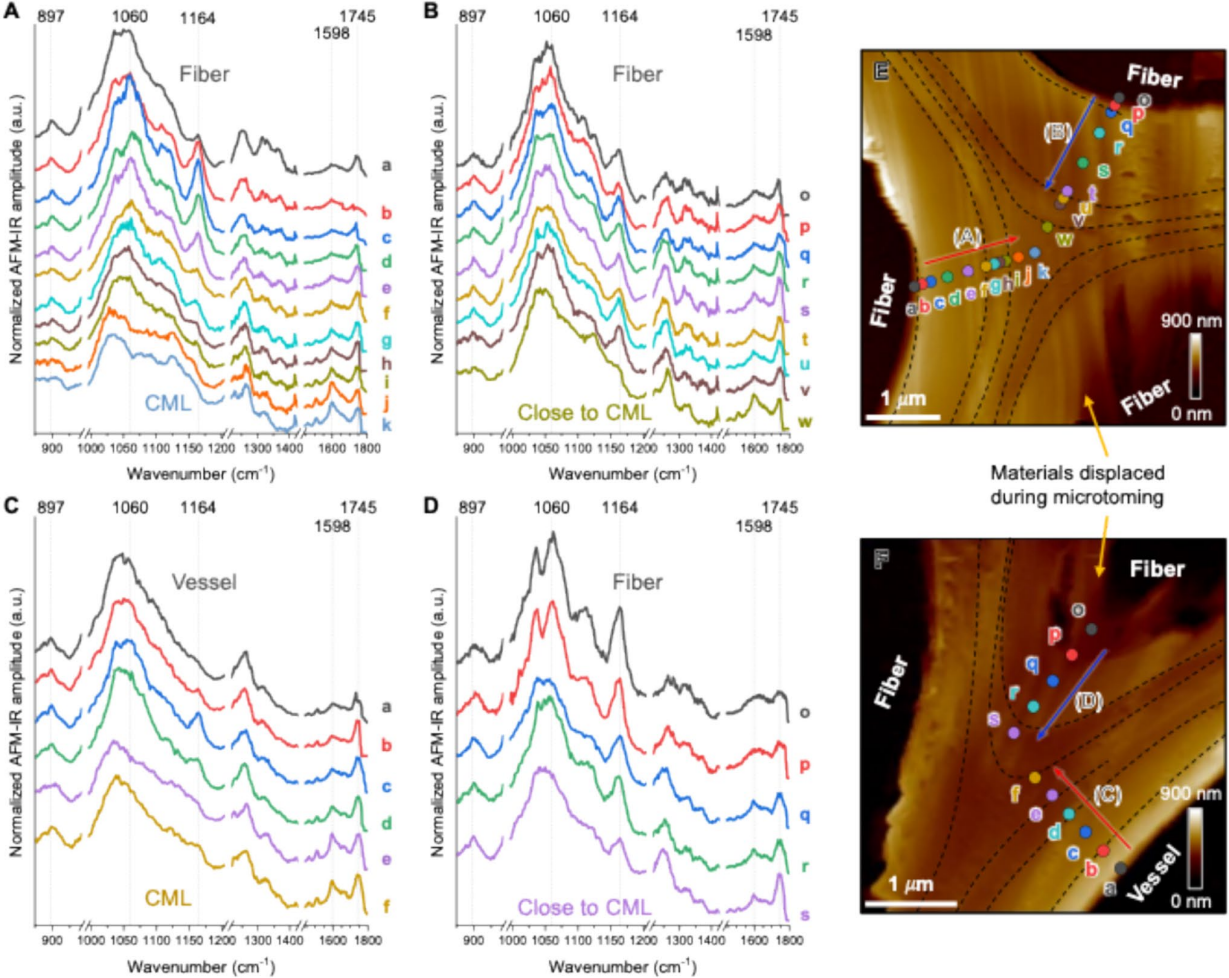
**A**-**D** AFM-IR analysis across poplar xylem cell walls. **A, B, D** Spectra collected from fiber cells toward the three-cell wall junction. **C** Spectra collected from a vessel cell toward the three-cell junction. **E, F** Topography images showing the locations where the AFM-IR spectra were collected. Labeled markers are color-coded to match the corresponding spectra. Dashed lines indicate suggested boundaries delineating sublayers, as identified through AFM-IR analysis

AFM-IR intensity ratio maps enhance sublayer differentiation by reducing experimental artifacts that affect raw intensity maps. By taking ratios between two wavenumbers at each pixel, shared artifacts can be canceled out, leading to improved sublayer contrast. Figure 3 shows the AFM-IR amplitude ratio maps at the representative wavenumbers normalized to the 1060 cm^−1^ peak intensity; this peak was selected as an internal reference because most spectra exhibited relatively strong spectral bands centered at ~ 1060 cm^−1^ (see Fig. 4). Additional AFM-IR ratio maps constructed using alternative band combinations, including cellulose-, lignin-, and hemicellulose-associated peaks, are provided in Fig. S1 and Fig. S2. A broad band with maxima in the range of 1034–1060 cm^−1^ is a common feature of most carbohydrate compounds (Lee et al. 2015; Agarwal et al. 2021; Kesari et al. 2021). As expected, the vertical stripe patterns caused by surface topography—visible in the amplitude maps (Fig. 2E,G,I)—are less pronounced in the ratio maps (Fig. 3A-C). In addition, new local contrasts that were not obvious in the raw intensity maps appear more prominent in the ratio maps, particularly in regions marked by arrows in Fig. 3D-F. These localized contrasts suggest additional fine-scale heterogeneity that is not readily resolved in the raw intensity maps.

#### Principal component analysis (PCA) of local AFM-IR spectra across cell wall sublayers

To further explore the capability of AFM-IR to differentiate the chemical composition in sublayers, full spectra over the 800–1800 cm⁻^1^ region were collected at multiple locations across the fiber and vessel cell walls. The AFM-IR spectra in Fig. 4 exhibit typical IR spectral features of plant cell walls. As mentioned before, the peak at 1164 cm⁻^1^ can be assigned to cellulose although non-cellulosic components can contribute to the baseline intensity at this position (Makarem et al. 2019). The peak near 900 cm⁻^1^ can also be attributed to cellulose (Agarwal and Ralph 1997; Lee et al. 2015; Coste et al. 2021). The peak at 1745 cm^−1^ is associated with methylated pectin and hemicellulose. The peaks at 1226 cm⁻^1^, 1508 cm⁻^1^, and 1598 cm⁻^1^ are putatively assigned to lignin (Agarwal and Ralph 1997; Kesari et al. 2021; Nepal et al. 2023). As shown in Fig. 4A, the relative intensities of these characteristic bands vary across spatial locations, indicating chemical heterogeneity that is not readily captured by single-pick analysis alone. To better classify wall sublayer and objectively assess spectral variation across locations, principal component analysis (PCA) was applied to the spectral dataset. The PCA results of the a-k spectra in Fig. 4A are shown in Fig. S3A in the Supplementary Information. Based on the clustering of data in the PCA score plot, the spectral features along the trajectory from points ‘a’ to ‘k’ in Fig. 4E can tentatively be attributed to different subsections: (a) = S3, (c, d, e) = S2, (f, g, h, i) = S1, and (j, k) = CML.

Compared to the spectra in Fig. 4A, the spectra in Fig. 4B exhibit smaller variation in spectral features, suggesting more compositional similarity across sampling points. This lower spectral variance is also reflected in the PCA score plot (Fig. S3B), where data points are more tightly clustered with smaller score values. Nonetheless, by examining the plot more closely, distinct groupings can still be resolved. The PCA score plot (Fig. S3B) shows that spectra from nearby locations tend to cluster together, leading to the following groupings inferred: (o, p, q) = S3, (r, s) = S2, (t, u, v) = S1, and (w) = CML. These groupings correspond well with the physical sampling locations shown in Fig. 4E.

AFM-IR spectra from walls adjacent to vessel and fiber cells (Fig. 4C,D) were also analyzed. While the limited number of sampling points in these regions reduces the statistical reliability of PCA (Fig. S3C,D), general spectral trends suggest plausible layer assignments: (a, b) = S3, (c) = S2, (d) = S1, and (e, f) = CML. The PCA analysis of the AFM-IR spectra in Fig. 4D could not be used for grouping as there are only five data points (Fig. S3D); the low score values as well as scattered score plots also suggest further interpretation may not be reliable. Nonetheless, based on a combined analysis of AFM-IR spectra, amplitude ratio maps, and PCA, delineation of sublayers of the cross-sections analyzed in Fig. 4E,F can be made, which are marked with dashed lines. These boundaries are tentative and should be refined with higher sampling density.

#### SFG microscopy analysis of hybrid poplar xylem

##### Hyperspectral SFG imaging of transversely cross-sectioned xylem

**Fig. 5:**
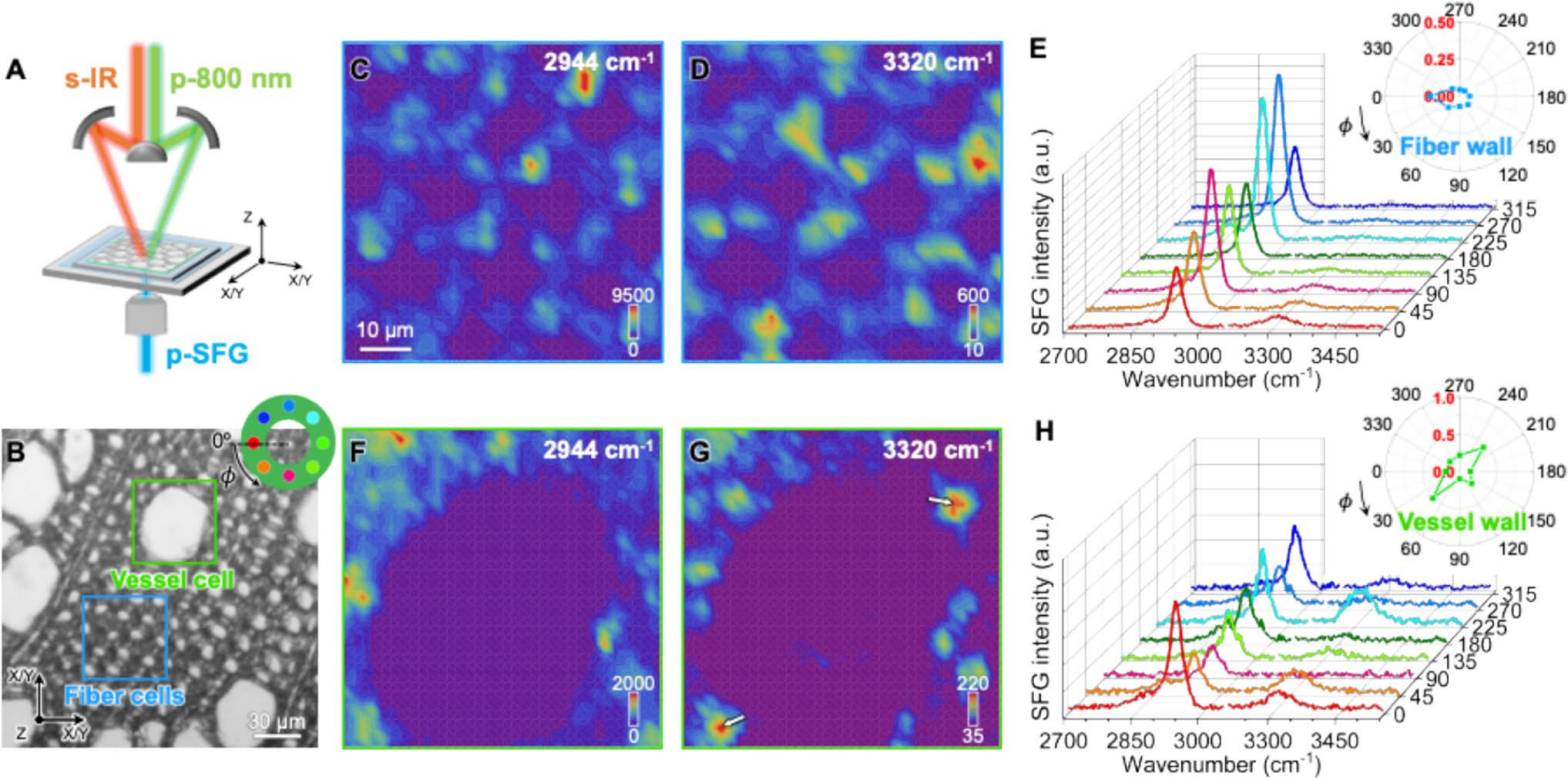
Representative SFG microscopy analysis of transversely cross-sectioned poplar xylem. **A** Schematic of the transmission SFG microscopy setup. The pps polarization combination was used for measurements. **B** Transversely cross-sectioned poplar xylem, with boxed areas indicating the regions selected for hyperspectral SFG imaging of fiber cells (blue) and a vessel cell (green). The inset illustrates the eight locations surrounding individual cells used to study microfibril angle (MFA) in **E** and **H**. **C**, **D**, **F**, **G** pps-SFG hyperspectral images (60 μm × 60 μm) of the fiber cell (**C**, **D**) and vessel cell (**F**, **G**) regions at 2944 cm^−1^ (**C**, **F**) and 3320 cm^−1^ (**D**, **G**). **E**, **H** pps-SFG spectra from the eight selected locations around a single fiber (**E**) and vessel (**H**) cell. The insets show polar plots of the 3320 cm^−1^/2944 cm^−1^ intensity ratios from the eight locations. A total of 6–8 fiber cell walls and three vessel cell walls from three transverse sections were analyzed, with additional replicates provided in Fig. S4-6. Scale bar in **C** applies to **D**, **F**, **G**

To investigate the mesoscale organization of cellulose microfibrils within the fiber and vessel cell walls of poplar xylem tissue, hyperspectral SFG imaging was employed. SFG microscopy system provides a few micrometer-scale local information by acquiring full vibrational spectra at each pixel, with a step distance of 2 μm. Figure 5 presents the intensity maps of cellulose-specific SFG signals extracted from hyperspectral images of transversely cross-sectioned fiber and vessel regions. Vessel cells are large (~ 50 μm in diameter), while fiber cells are smaller (~ 10 μm in diameter). In both cell types, the SFG hyperspectral images (Fig. 5C,D,F,G) show that the OH stretch signal at 3320 cm⁻^1^ is markedly lower than the CH stretch signal at 2944 cm⁻^1^, and the local variation is larger for the vessel wall than the fiber wall.

For analysis of cellulose microfibril orientation, the OH/CH intensity ratio is more informative than individual peak intensities, as it factors out the intensity variation from the amount of crystalline cellulose. The ratio is known to be highly sensitive to the orientation of CMFs relative to the laser beam polarizations in the SFG system, and has been used to estimate the azimuth and tilt angles of CMFs with respect to the laser incident plane and sample stage (Chen et al. 2017; Huang et al. 2018; Lee et al. 2023; Choi et al. 2023). As the S2 layer is the thickest among the secondary cell wall sublayers, it is assumed that the majority of the SFG signal originates from this layer. In the fiber cell region (Fig. 5E), the OH/CH ratios around the cell wall perimeter are quite uniform and remain low (within the 0.1–0.2 range). This trend was consistently observed across multiple fiber cells in different replicates (Fig. S4F-M, Fig. S5G-L, Fig. S6G-L). In contrast, the vessel cell region (Fig. 5H) shows this trend: larger local variation in the OH/CH intensity ratio around the single cell wall perimeter. The corresponding OH/CH ratio polar plot shows slight anisotropy; however, this trend was less evident in other vessel cell replicates (Fig. S5M, Fig. S6M).

##### SFG microscopy analysis of longitudinally cross-sectioned xylem

**Fig. 6:**
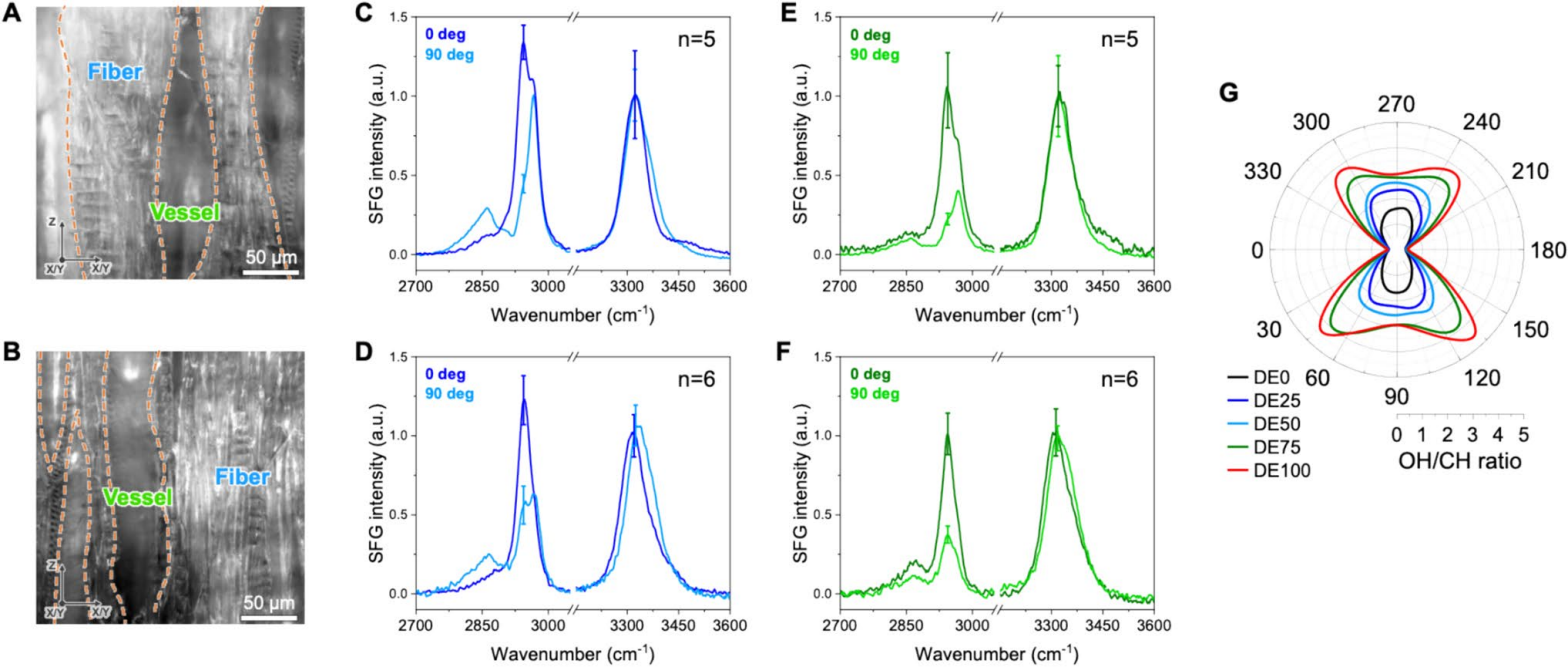
SFG microscopy analysis of two longitudinal cross-sections of poplar xylem tissue. **A**, **B** Micrographs of two longitudinal sections, showing fiber and vessel cell regions. **C**-**F** Averaged SFG spectra collected from multiple fiber (**C**,** D**) and vessel (**E**, **F**) cell wall locations at 0° and 90° sample orientations (*n* = 5 or 6, as indicated). The 0° orientation corresponds to the longitudinal (stem) axis being parallel to the laser incident plane. **G** Simulated polar plots of the pps-SFG OH/CH intensity ratio (2944 cm^−1^/3320 cm^−1^) with various directional excess (DE) values: 0%, 25%, 50%, and 100%. Simulation parameters: d = 4 nm, Δl = 5 nm, θ = 90° with σ_θ_ = σ_ϕ_ = 20°, ψ = random. Details of the simulation method are described in previous literature (Choi et al. 2022). The angular distribution input was estimated based on the FWHM of the wide-angle X-ray scattering rocking curve shown in Fig. S7

SFG analysis of longitudinally cross-sectioned xylem tissue (Fig. 1D,G) was further performed to probe CMF orientation. Figure 6 shows SFG spectra from fiber and vessel cell wall regions, collected at two orientations: i.e., the longitudinal (stem) axis is parallel (0°) and perpendicular (90°) to the laser incidence plane. To facilitate the analysis of changes in relative intensities between CH (2944 cm⁻^1^) and OH (3320 cm⁻^1^) peaks, the collected spectra were normalized to the 3320 cm⁻^1^ peak and then averaged. At 0°, the 2944 cm⁻^1^ intensity is comparable to or slightly greater than the 3320 cm⁻^1^ intensity in both fiber and vessel cell walls (i.e., the 3320 cm⁻^1^/2944 cm⁻^1^ ratio is close to 1). After rotating the sample by 90°, this ratio increases significantly: to ~ 2 in fiber walls and ~ 3 in vessel walls. This change in the OH/CH ratio with sample orientation was observed in both cell types, and the experimental trend is qualitatively consistent with the simulated azimuth-dependent *pps*-SFG polar plots in Fig. 6G.

## Discussion

### Limitations of AFM-IR amplitude maps and advantage of ratio maps

AFM-IR provides chemical information at nanoscale spatial resolution, far better than the conventional imaging techniques based on focusing IR beams with an objective lens; thus, it enables the detection of wall sublayers and variations in chemical composition in plant cell walls (Coste et al. 2021; Kesari et al. 2021). This capability is particularly valuable for investigating chemically heterogeneous secondary cell walls in woody tissues. The strong 1594 cm^−1^ intensity at the center of the three-cell junctions is consistent with high lignin content in the compound middle lamella (CML) region. In plants that undergo secondary cell growth and have thick xylem woody tissue, such as the two-year-old, field-grown poplar in this study, lignin is distributed throughout the wall but is particularly enriched in the CML region, which serves as a glue between adjacent cells (Whiting and Goring 1983; Kesari et al. 2021).

However, it should be noted that the AFM-IR intensities do not directly reflect the sublayer compositional variations. In the 1164 cm^−1^ intensity map (Fig. 2E,F), the thick layers that are presumed to correspond to the S2 layer (left-side wall in Fig. 2E and bottom-right wall in Fig. 2F) appear less pronounced even though cellulose content in the S2 layer is expected to be high (Koshani et al. 2025). This must be associated with detection artifacts inherently involved in contact-mode AFM-IR. It should be noted that AFM-IR does not measure the IR absorption directly; instead, it detects surface height changes caused by thermal expansion of the subsurface region upon IR absorption. This process can be influenced by factors such as the efficiency of thermal dissipation of a portion of the absorbed IR energy, subsurface structures including network connectivity, anisotropy, and porosity, as well as dynamic coupling between the probe and surface (Lin et al. 2022, 2024). For example, differences in CMF packing and matrix composition across layers could alter thermal expansion behavior. The vertical stripe patterns following topographic contours (red arrows in Fig. 2C,E) may also arise from such artifacts. Thus, relying solely on the raw intensity maps may be misleading; alternatively, analyzing relative intensity ratios among characteristic peaks would provide more physically meaningful information to reveal relative chemical differences between sublayers.

In the AFM-IR amplitude ratio maps, the localized contrasts marked by arrows in Fig. 3 (see also Fig. S1 and Fig. S2) may correspond to transition layers, similar to those observed in a photo-induced force microscopy (PiFM) study of Norway spruce by Kesari et al. (2021). These observations suggest that ratio-based AFM-IR analysis can provide additional information beyond that accessible from raw amplitude maps alone, particularly for resolving subtle compositional variations within complex secondary cell wall architectures.

However, it should also be noted that the choice of the reference peak can influence the differentiation of the chemical contrast in AFM-IR imaging. For example, if the 1060 cm⁻^1^ peak is used as the denominator, the resulting ratio could be misinterpreted as a higher cellulose signal in the compound middle lamella (CML) region (Fig. 3A). The AFM-IR spectra in Fig. 4 support this concern, showing that the peak intensities vary across positions, and 1060 cm⁻^1^ is not always the spectral maximum. To address this ambiguity, complementary ratio maps between distinct biochemical bands are presented in Fig. S1 and Fig. S2: cellulose/lignin ratio (1164 cm^−1^/1594 cm^−1^), cellulose/hemicellulose ratio (1164 cm^−1^/1745 cm^−1^), and lignin/hemicellulose (1594 cm^−1^/1745 cm^−1^). These maps may offer a clearer representation of the relative compositions of cellulose, lignin, and hemicellulose across different cell wall regions in hybrid poplar.

### Sublayer heterogeneity revealed by AFM-IR spectra and PCA

The combined AFM-IR spectra and PCA analysis provide insight into the chemical heterogeneity of the hybrid poplar xylem secondary cell wall sublayers at nanoscale. The spectral features and their progression from lumen-side regions (e.g., points ‘a’ and ‘o’) to the shared CML (‘k’ and ‘w’) are different across the neighboring walls (Fig. 4A,B,E). This suggests that sublayer distribution is different even between two adjacent fiber cell walls that share the same middle lamella. The primary cell wall (PCW) is too thin and therefore may not have been captured during the stepwise change of the spectral analysis location. Even if the AFM tip was placed right at the PCW position, its spectral features might be convoluted by the contributions from neighboring S1 and CML regions.

Notably, the vessel-facing S2 spectrum (‘c’ in Fig. 4C) exhibits a lower intensity at 1164 cm⁻^1^ compared to the fiber-facing cell wall spectra (‘o’- ‘r’ in Fig. 4D), suggesting a lower cellulose content in the vessel-facing cell walls. This trend is also supported by the AFM-IR amplitude map at 1164 cm⁻^1^ in Fig. 2F and the amplitude ratio maps in Fig. S2A,C. Such compositional differences may reflect biological differentiation in cell function, as fiber cells provide structural support, whereas vessel cells specialize in water conduction. Such functional divergence likely translates into distinct wall composition and layering.

The tentative delineation of sublayers based on a combined analysis of AFM-IR spectra, amplitude ratio maps, and PCA does not show clear representation of the classical S1-S3 sublayers, suggesting that the hybrid poplar xylem secondary cell wall (SCW) exhibits more complex heterogeneity. A previous high-resolution PiFM study of Norway spruce revealed additional transition layers and highly lignified regions on the scale of ~ 20 nm between classical S1–S3 sublayers (Kesari et al. 2021). A similar phenomenon may be present here; for example, points ‘b’ and ‘c’ in Fig. 4A show high 1164 cm⁻^1^ and low 1745 cm⁻^1^ intensity, distinct from adjacent S2 regions (d, e), potentially indicating a transitional sublayer. This interpretation is supported by PCA score separation (Fig. S3A). Additionally, points ‘o’ and ‘p’ in Fig. 4D may correspond to a gelatinous layer (G-layer), which is known to form in tension wood under mechanical stress (Kafle et al. 2014; Ghislain et al. 2019). G-layers are typically rich in cellulose and exhibit larger cellulose crystallite sizes (Wada et al. 1995; Jonasson et al. 2021). Although the poplar trees analyzed in this study were grown under upright conditions, field exposure to wind or minor leaning could have triggered localized G-layer development. While limited sampling density may contribute, the fact that the points in the PCA score plots in Fig. S3 do not cluster into four distinct groups (i.e., CML, S1, S2, S3) suggest chemical heterogeneity and the presence of transitional layers.

### CMF orientation from SFG imaging of transverse sections

The vibrational SFG spectroscopy is a nonlinear optical spectroscopy technique capable of probing cellulose crystallinity and orientation studies in hydrated, intact plant cell walls (Makarem et al. 2019; Koshani et al. 2025; Lee et al. 2025). The local intensity variations in the SFG intensity maps at 2944 cm^−1^ (Fig. 5C,F) likely reflect differences in crystalline cellulose content, as SFG selectively probes crystalline cellulose in plant cell walls (Barnette et al. 2011; Makarem et al. 2019; Lee et al. 2025). This spatial variation in cellulose is consistent with the AFM-IR observations in Fig. 4A,B, where the intensity associated with cellulose at 1164 cm^−1^ varied even between adjacent fiber cell walls sharing the same middle lamella, suggesting localized heterogeneity in cellulose content.

Recent advances in theoretical SFG intensity calculations enable the estimation of MFA in individual walls (Choi et al. 2022, 2023). Accordingly, the mesoscale organization of CMF assemblies at each pixel can be inferred through spectral interpretation. By analyzing the spectra from walls surrounding individual cells, the MFA in fiber and vessel walls can be estimated. The small and relatively uniform OH/CH intensity ratio observed around the fiber cell perimeter (Fig. 5E) indicates vertically aligned cellulose microfibrils with near-zero tilt angle (Choi et al. 2023), as illustrated in Fig. 1B-D. Such uniformity implies minimal variation in microfibril orientation along the cell perimeter, meaning that the CMF orientation with respect to the laser incident plane (ZX or ZY plane in Fig. 5A and Fig. 1C) does not change in the cell walls around the cell perimeter (Choi et al. 2023).

In contrast, when there is a nonzero tilt angle, as shown in Fig. 1E-G, the CMF orientation with respect to the laser incident plane varies around the cell, resulting in OH/CH ratio changes around the cell perimeter depending on the azimuth angle, which is the angle between the X/Y-axis and the CMF within the XY plane in Fig. 1F (Chen et al. 2017; Choi et al. 2023). The larger OH/CH intensity ratio variation around the vessel cell perimeter reflects this behavior. The corresponding OH/CH ratio polar plot (Fig. 5H) shows slight anisotropy, indicating that the CMFs deviate from the stem axis by a certain tilt angle, as illustrated in Fig. 1F. This qualitative observation aligns with previous polarized light microscopy studies, which reported that MFAs in vessel cell walls are higher than those in fiber cell walls in the secondary xylem of Japanese hardwood (Kita et al. 2023). However, the less pronounced anisotropy observed in other vessel cell replicates (Fig. S5M, Fig. S6M) suggest that if a tilt exists, it is likely small or comparable to that of fiber cell walls.

### CMF orientation and polarity from SFG analysis of longitudinal sections

SFG analysis of longitudinally cross-sectioned samples provides additional insight into CMF orientation, particularly when CMFs are nearly aligned along the stem axis with minimal tilt angle (Choi et al. 2023). The pronounced change in the OH/CH ratio with sample orientation in both cell types (Fig. 6C-F) indicates a high degree of CMF alignment along the longitudinal (stem) axis and is consistent with the near-zero microfibril tilt angle found in the SFG analysis of transversely cross-sectioned samples in Fig. 5 (Choi et al. 2023). In *pps*-SFG, when CMFs are aligned parallel to the laser incidence plane, the OH/CH intensity ratio is low; when perpendicular, the ratio increases (Chen et al. 2017; Huang et al. 2018). Accordingly, the experimentally observed OH/CH ratio changes reflect the expected response of highly aligned CMFs and are further supported by the simulated azimuth-dependent *pps*-SFG polar plots in Fig. 6G.

When CMFs are highly aligned, the bundled CMFs can exhibit varying polarity because individual CMFs at nanoscale have polarity; i.e., they can have unidirectional, bidirectional, or intermediate polarity alignment (Chae et al. 2021; Choi et al. 2023; Lee et al. 2025). This polarity distribution at meso-/microscale can be described by the directional excess (DE) parameter (Choi et al. 2022); when they are perfectly unidirectional, DE is 100, and when they are bidirectional—meaning that 50% of CMFs are oriented opposite to the other 50%—DE is 0 (Choi et al. 2022). Although the agreement between the simulated OH/CH ratio profiles in Fig. 6G and the experimental trend is only qualitative rather than quantitative, the simulation results and the observed OH/CH ratio changes upon 90° sample rotation suggest that CMFs in fiber walls exhibit a lower directional excess (DE)—i.e., they are more bidirectionally oriented—compared to those in vessel walls.

### Comparison with *Arabidopsis thaliana* stem

**Fig. 7:**
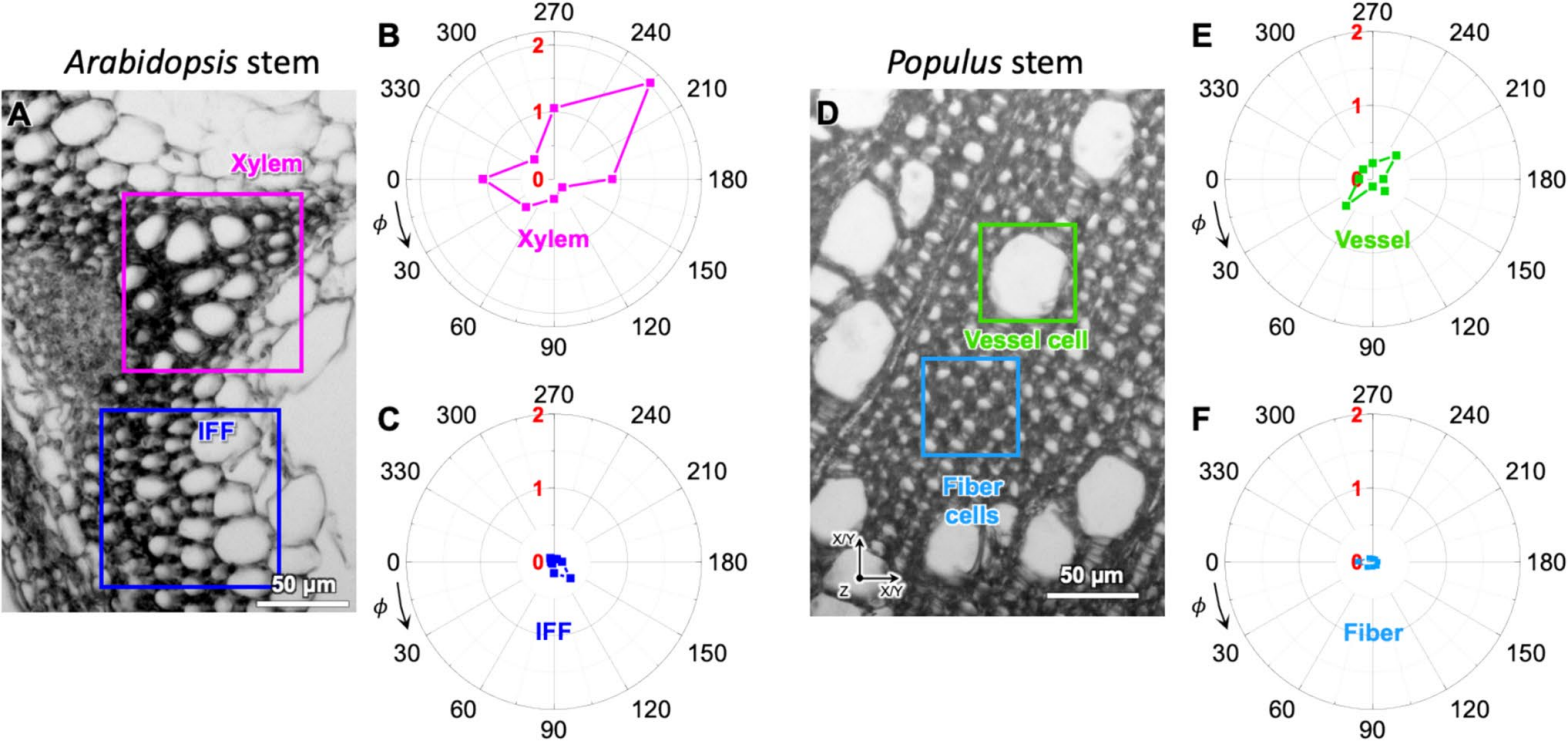
Comparison of pps-SFG intensity ratio polar plots (3320 cm⁻1/2944 cm⁻1) from two-year-old poplar and 8-week-old *Arabidopsis* stems. **A** Transverse section of *Arabidopsis* stem with the xylem region (magenta) and interfascicular fiber (IFF, navy blue) region labeled. **B**, **C** Corresponding polar plots for the xylem (**B**) and IFF (**C**) regions. **D** Transverse section of poplar stem with vessel (green) and fiber (blue) regions marked. **E**, **F** Polar plots of the 3320/2944 cm⁻1 intensity ratio for the vessel (**E**) and fiber (**F**) regions. **A-C** were replotted with permission from (Choi et al. 2023)

Figure 7 compares the MFA of CMFs in fiber and vessel walls in the xylem tissue of two-year-old poplar (a woody plant) analyzed in this study with that in interfascicular fiber (IFF) and xylem cell walls in the stem of eight-week-old *Arabidopsis thaliana* (an annual herbaceous plant). It should be noted that the vessel cells in poplar xylem tissue and the xylem cells in *Arabidopsis* stems should not be confused, although both serve a similar water-conducting function. The previous MFA study of *Arabidopsis* stems reported tilt angles of approximately 5° for IFF and 19.9° for xylem cell walls (Choi et al. 2023). In that study, it was proposed that the small tilt angle in IFF cell walls reflects their role in mechanical support, while the larger tilt in xylem cell walls corresponds to their role in water transport (Choi et al. 2023). The vertically aligned CMFs in IFF cell walls can better support body weight because the load-bearing capacity of cell walls is maximized along the fiber direction (Daniel 2007). In contrast, a more tilted or spiral CMF arrangement in xylem walls may help resist to wall collapse under the negative pressures generated by evapotranspiration of water at the leaf (Turner and Somerville 1997), as suggested by analogies with fiber-reinforced composite pressure vessels with different fiber winding angles (Parnas and Katırcı 2002).

The polar plots of OH/CH intensity ratios surrounding IFF and xylem cells in *Arabidopsis* show a two-lobed distribution in the xylem (Fig. 7B) and a radially symmetric, near-zero OH/CH ratio pattern in the IFF (Fig. 7C). Similar polar plot patterns were observed in the vessel (Fig. 7E) and fiber (Fig. 7F) cell walls in poplar xylem tissue. The polar plots of fiber and vessel cells in poplar resemble those of the IFF and xylem cells in *Arabidopsis* stems, respectively. Interestingly, the functional distinction between fiber and vessel cells in poplar—mechanical and hydraulic support, respectively—is consistent with these structural observations (Woodrum et al. 2003; Pratt et al. 2007; Plavcová et al. 2013). The smaller OH/CH ratios in poplar xylem tissue compared to *Arabidopsis* stems suggest a smaller MFA, i.e., a higher degree of vertical CMF alignment along the stem axis. This enhanced alignment is likely due to the thicker secondary cell walls in poplar, which demand greater mechanical strength to support the load-bearing requirements of a woody plant species.

In summary, fiber and vessel cell walls in a two-year-old, field-grown hybrid poplar (*P. alba* × *P. glandulosa*) stem were investigated using photothermal AFM-IR and SFG microscopy and compared with previous SFG microscopy study of eight-week-old *Arabidopsis thaliana*. The AFM-IR results revealed chemical heterogeneity even between adjacent cell walls sharing the same junction, as well as potential localized transitional zones  beyond the canonical S1, S2, and S3 sublayers of the secondary cell wall. While such transitional layers have been described in wood anatomy, their detection in hybrid poplar through AFM-IR highlights its capability to probe nanoscale chemical variation that is not readily accessible by conventional methods. The SFG analysis indicated that the MFA in fiber cell walls is close to zero, reflecting highly aligned cellulose microfibrils along the longitudinal axis of the stem. In contrast, the MFA in vessel cell walls showed a slight deviation from zero. These findings support the view that plant cell walls are produced with three-dimensional architectures adapted to their biological functions: mechanical support for fiber cells and hydraulic conduction for vessel cells. This study demonstrates the complementary use of AFM-IR and SFG microscopy for nanoscale chemical imaging and meso-to-microscale CMF orientation analysis of plant cell walls. Although based on one representative tree, this approach can provide a new opportunity to study how cellulose microfibril orientation and chemical composition vary by cell type and wall layer, advancing our understanding of cell wall structure–function relationships in woody plants.

## Supplementary Information

Below is the link to the electronic supplementary material.

Supplementary file1 (DOCX 9041 KB)

## Data Availability

All data in this study are available from the corresponding author upon reasonable request.

## Abbreviations

*AFM-IR*: Atomic force microscopy-infrared spectroscopy
*CMF*: Cellulose microfibril
*MFA*: Microfibril angle
*PCA*: Principal component analysis
*SFG*: Sum frequency generation
