# Supplementary material for: Microfibril orientation and compositional heterogeneity in fiber and vessel cell walls of poplar xylem studied by AFM-IR and SFG spectroscopy
Source: Planta. 2026 Feb 19;263(3):77. doi: 10.1007/s00425-026-04947-7 (PMC12920761; doi:10.1007/s00425-026-04947-7)
Supplement: Supplementary file 1 — Supplementary file1 (DOCX 9041 KB) [file 425_2026_4947_MOESM1_ESM.docx]

**Supplementary Information for**

**Microfibril orientation and compositional heterogeneity in fiber and vessel cell walls of poplar xylem studied by AFM-IR and SFG spectroscopy**

Jongcheol Lee^1,*^, Juseok Choi^1^, Yen-Ting Lin^1^, Fangxin Qian^1^, Botong Tong^2^, Quanzi Li^3^, and Seong H. Kim^1,*^

^1^*Department of Chemical Engineering and Materials Research Institute, Pennsylvania State University, University Park, Pennsylvania 16802, USA*;

^2^*Institute of Industrial Crops, Heilongjiang Academy of Agricultural Sciences, Harbin 150086, China*;

^3^*National Key Laboratory for Development and Utilization of Forest Food Resources, Zhejiang A&F University, Hangzhou 311300, China*

^*^corresponding authors; Email: jongcheol1422@gmail.com, shk10@psu.edu


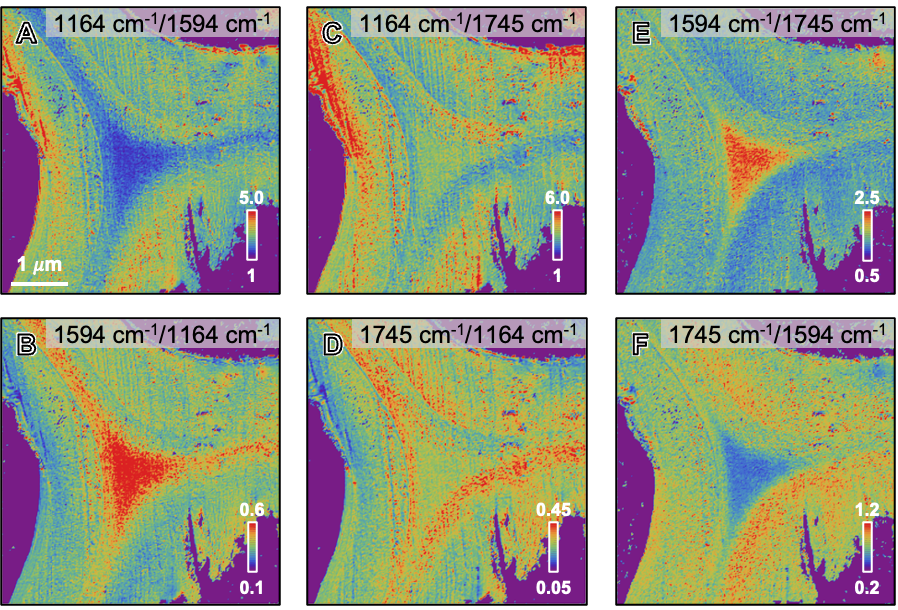


**Fig. S1** Additional AFM-IR amplitude ratio maps of cross-sectioned walls surrounded by fiber cells. Wavenumber pairs used for each ratio are indicated in the top-right corner of each panel.

**
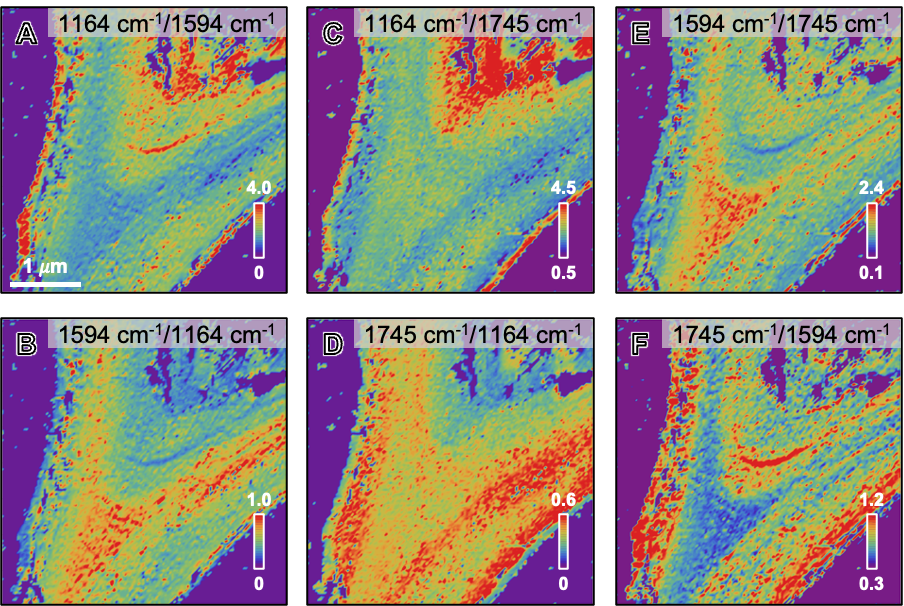
**

**Fig. S2** Additional AFM-IR amplitude ratio maps of cell wall adjacent to vessel and fiber cells. Wavenumber pairs used for each ratio are indicated in the top-right corner of each panel.


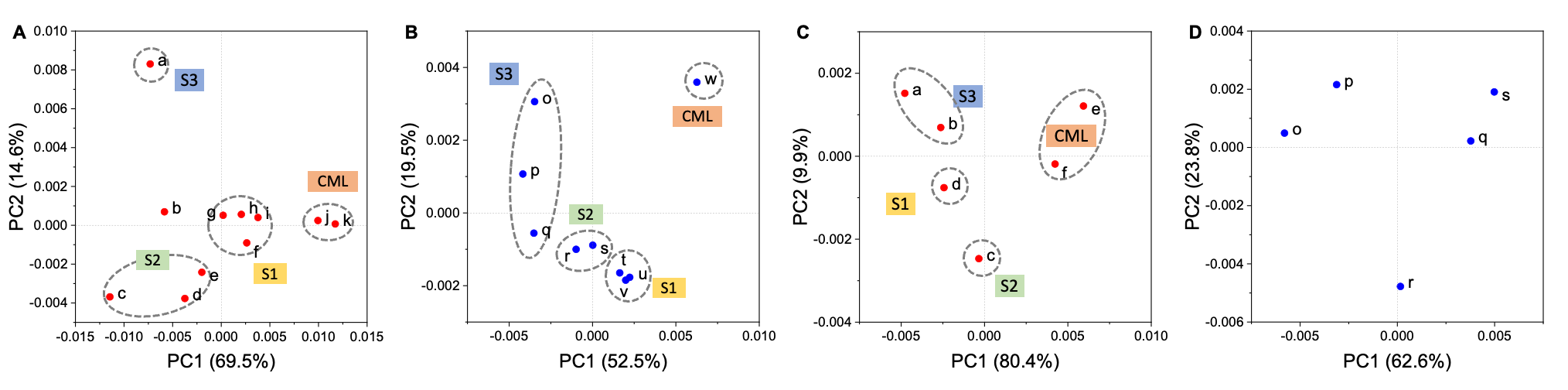


**Fig. S3** Principal component analysis (PCA) score plots of AFM-IR spectra collected across fiber and vessel cell walls shown in Fig. 4. **A-D** corresponds to the Fig.4A-D, respectively. Labels in the score plots correspond to the sampling points marked in Fig. 4: ‘a’-‘k’ in A and ‘o’-‘w’ in B from Fig. 4E, and ‘a’-‘f’ in C and ‘o’-‘s’ in D from Fig. 4F. The score plots distinguish secondary wall sublayers, including S1, S2, S3, and compound middle lamella (CML) regions.

**Fig. S4** SFG microscopy analysis of hybrid poplar tree xylem tissue (sample 1). **A** Optical micrograph of a transversely cross-sectioned poplar xylem. Boxed regions indicate fiber cells (blue) and a vessel cell (green), selected for SFG hyperspectral imaging. **B-E** pps-SFG hyperspectral images (60 μm × 60 μm) collected at 2944 cm^-1^ (**B**, **D**) and 3320 cm^-1^ (**C**, **E**) for the vessel and fiber cell regions. **F-N** Polar plots of the 3320 cm^-1^/2944 cm^-1^ ratio from eight locations surrounding each cell. A total of eight fiber cells (**F-M**) and one vessel cell (**N**) were analyzed.

**Fig. S5** SFG microscopy analysis of hybrid poplar tree xylem tissue (sample 2). **A**, **B** Optical micrographs of transversely sectioned xylem showing boxed regions selected for fiber cells (blue) and a vessel cell (green). **C–F** pps-SFG hyperspectral images (60 μm × 60 μm) acquired at 2944 cm⁻¹ (**C**, **E**) and 3320 cm⁻¹ (**D**, **F**) for the vessel and fiber regions, respectively. **G–M** Polar plots of the 3320 cm⁻¹/2944 cm⁻¹ ratio from eight locations around each cell. A total of six fiber cells (**G–L**) and one vessel cell (**M**) were analyzed.

**Fig. S6** SFG microscopy analysis of hybrid poplar tree xylem tissue (sample 3). **A**, **B** Optical micrographs of transversely sectioned xylem with boxed regions selected for fiber cells (blue) and a vessel cell (green) SFG imaging. **C–F** pps-SFG hyperspectral images (60 μm × 60 μm) acquired at 2944 cm⁻¹ (**C**, **E**) and 3320 cm⁻¹ (**D**, **F**) for the vessel and fiber regions, respectively. **G–M** Polar plots of the 3320 cm⁻¹/2944 cm⁻¹ ratio from eight locations around each cell. A total of six fiber cells (**G–L**) and one vessel cell (**M**) were analyzed.

**Fig. S7** Wide-angle X-ray scattering (WAXS) analysis of poplar xylem. **A** 2D X-ray scattering pattern of a longitudinally sectioned poplar xylem sample, showing anisotropic diffraction features indicative of cellulose microfibril alignment. **B** X-ray diffraction (XRD) pattern obtained by azimuthal integration of the 2D scattering image, displaying characteristic cellulose peaks. **C** Azimuthal intensity profile (rocking curve) at 2θ = 22°, used to assess the angular distribution of aligned microfibrils. The full width at half maximum (FWHM) of 45.6° reflects the spread in microfibril orientation and was used to estimate the angular distribution (σ) in SFG simulations (Fig. 6G). The standard deviation ($\sigma$) was calculated to be ~20° using $\sigma=FWHM/(2\sqrt{2ln2})$.
